# Ocular Decompression Retinopathy Following Canaloplasty for Primary Open Angle Glaucoma: A Case Report

**DOI:** 10.1097/MD.0000000000002907

**Published:** 2016-03-07

**Authors:** Gai-yun Li, Samer Alantaree, Jun-ming Wang, Hong Zhang

**Affiliations:** From the Department of Ophthalmology, Tongji Hospital, Tongji Medical College, Huazhong University of Science and Technology, Wuhan, Hubei Province, China.

## Abstract

Ocular decompression retinopathy (ODR), a rare postoperative complication following glaucoma surgery, is characterized by the transient appearance of scattered retinal hemorrhages.

Here, we present a unique case of ODR in a patient with primary open angle glaucoma who underwent canaloplasty. A 31-year-old male patient presented with an intraocular pressure (IOP) of 60 mm Hg in the right eye. The IOP remained over 40 mm Hg, even when treated with maximum tolerated antiglaucoma medication. Canaloplasty drastically lowered IOP in the right eye from 40 to 7 mm Hg. However, fundus examination revealed ODR after surgery. The patient was treated with tobramycin and dexamethasone. Three months after canaloplasty, IOP remained in control at 16 mm Hg and all retinal hemorrhages had completely resolved.

This case demonstrates that ODR can occur following canaloplasty and physicians should be aware of this potential complication in patients with severely elevated IOP. Sufficiently lowering IOP before surgery and gradually decreasing IOP during surgery may prevent ODR from occurring.

## INTRODUCTION

Fechtner et al^1^ first reported the presence of retinal hemorrhages following trabeculectomy under the descriptive term of ocular decompression retinopathy (ODR). This condition is defined as a multifocal hemorrhagic retinopathy that occurs following acute lowering of intraocular pressure (IOP) that is not explained by another pathological process.^2^ Hemorrhages occur throughout the fundus and often have white centers. Visual acuity is generally unaffected.^1^ Although the pathological mechanism of ODR is not agreed upon, elevated IOP subsequent rapid IOP reduction seems to be associated with the condition. In addition to trabeculectomy, ODR has been observed following Ahmed valve implantation, peripheral iridotomy, anterior chamber paracentesis, deep sclerotomy, ExPRESS shunt implantation, and IOP-lowering medication use.^3–8^ Canaloplasty is a new bleb-independent glaucoma surgery that is an established treatment for POAG. By canulating, viscodilating, and stenting Schlemm canal (SC), aqueous humor outflow via the physiological collector system is facilitated.^9^ To the best of our knowledge, ODR following canaloplasty has not been previously reported in the literature.

## CONSENT

This study adhered to the tenets of the Declaration of Helsinki. Informed consent was signed by the patient for publication of this report and its related images.

## CASE PRESENTATION

A 31-year-old male patient presented with pain and decreased vision in the right eye that had begun 1 year earlier. Because all symptoms resolved with rest, so the patient did not seek medical attention. One month before presenting to our clinic, symptoms had dramatically worsened and were occasionally accompanied by nausea and vomiting. A review of the patient's medical history revealed that he had been diagnosed with a retinal detachment in the left eye at another eye clinic 2 years earlier. The patient chose not to undergo surgical repair of the retinal detachment because he was afraid to undergo surgery. Between that time and the first visit to our clinic, vision in the left eye had painlessly decreased to no light perception (NLP). The patient did not use antiglaucoma or anti-inflammatory medications and had no history of systemic disease.

At the time of presentation, best-corrected visual acuity (BCVA) was 20/200 (refractive correction of −4.75D) in the right eye and NLP in the left eye. The IOP was 60 mm Hg in the right eye and 9 mm Hg in the left eye. Ocular examination revealed mild corneal edema, clear lens, and a normal deep anterior chamber with a wide angle in the right eye and a normal anterior chamber and clear lens in the left eye. Fundus examination showed a vertical optic disc cup-to-disc ratio of 0.9 in the right eye and a long-standing retinal detachment associated with proliferative retinopathy in the left eye. Humphrey visual field testing (30-2) showed predominantly temporal inferior and superior visual field defects in the right eye. A diagnosis of POAG was given to the right eye based on clinical signs and symptoms.

**FIGURE 1:**
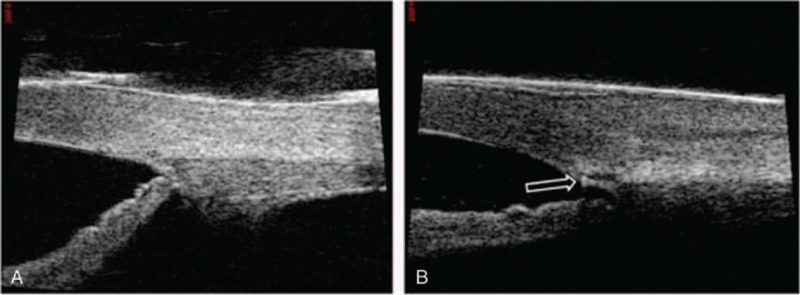
Ultrasound biomicroscopy (UBM) images of a 31-year-old man who underwent canaloplasty in the right eye. A, Preoperative UBM image of the anterior chamber at the 3 o’clock position. B, Postoperative UBM image of the anterior chamber at the 3 o’clock position. An enlarged Schlemm canal and a prolene suture within the canal (arrow) are easily seen.

The patient was admitted to the hospital and topical (travoprost, once a day; timolo, twice a day; brinzolamide, twice a day), oral (vinegaramin, 25 mg, 3 times a day), and intravenous (Mannitol 20%, twice a day) antiglaucoma agents were administered. Unfortunately, IOP in the right eye remained above 40 mm Hg despite administration of the maximum amount of medical therapy. Therefore, canaloplasty was performed on the right eye in a routine manner. After applying topical anesthesia, an incision was made in the conjunctiva and Tenon capsule and a 4 mm × 5 mm superficial scleral flap was created at the limbus at 12 o’clock. A deep flap was then created within the superficial flap margin. A temporal paracentesis was performed before SC was unroofed and a trabeculo-Descemet's window was formed. A flexible microcatheter (iTrack 250A; iScience Interventional, MenloPark, CA) was used to dilate the full circumference of the canal by injecting sodium hyaluronate 1.4% (Healon GV) during catheterization. Following complete circumferential dilation of SC, a 10-0 polypropylene tensioning suture was placed. The deep flap was excised and the superficial flap and conjunctiva were closed in a watertight manner. High-resolution ultrasound biomicroscopy (UBM) was used to assess SC and anterior segment angle morphology (Figure 1).

**FIGURE 2:**
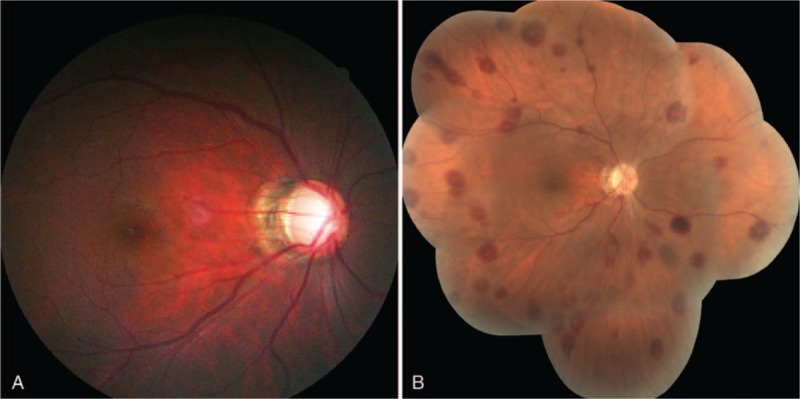
Color photographs of the right eye of a 31-year-old man diagnosed with primary open angle glaucoma. A, Fundus photograph of the right eye before canaloplasty was performed. There was no sign of retinal hemorrhage. B, Mosaic of retinal photograph taken 1 day following surgery. Multiple, diffuse, white-centered retinal hemorrhages are apparent.

One day after surgery, BCVA in the right eye was 20/100, the cornea was clear, and IOP was 7 mm Hg. Fundus examination revealed scattered white-centered retinal hemorrhages in the periphery and posterior pole (Figure 2). A mild choroidal detachment was detected in the right eye using UBM. Therefore, the patient was administered tobramycin and dexamethasone ophthalmic suspension every 2 hours. One week after surgery, the choroidal detachment had completely resolved, IOP had improved to 18 mm Hg, and retinal hemorrhages began to resolve. Three months after surgery, the BCVA in the right eye was 20/80 and IOP remained in control at 16 mm Hg with topical latanoprost 0.005% (1 drop per day, Pfizer Inc, New York, NY). Fundus examination showed complete resolution of all retinal hemorrhages.

## DISCUSSION

Ocular decompression retinopathy is an uncommon complication associated with surgical and medical procedures performed to treat glaucoma.^3–8^ The diffuse, multilevel hemorrhages seen in the fundus can mimic a central retinal vein occlusion. However, ODR does not present with venous dilation and delayed venous filling on fluorescein angiography.^10,11^ The differential diagnosis of ODR and other vascular disorders, including Valsalva retinopathy, Terson syndrome, and diabetic retinopathy, should be considered.

The pathogenesis of ODR is not fully understood, but several risk factors have been suggested.

Mandal et al^12^ theorized that the Valsalva maneuver performed during general anesthesia, which is known to increase retinal blood flow, might play a role in the development of ODR. However, in our case, surgery was performed under topical anesthesia. Additionally, our case is similar to 1 case reported by Kozobolis et al,^6^ which involved a case of ODR following deep sclerotomy performed under topical anesthesia. Together these cases suggest that general anesthesia is not a predisposing factor to the development of ODR.

Elevated IOP and the subsequent rapid IOP reduction seem to be associated with the development of ODR. Jung et al^13^ found that only preoperative IOP influenced the occurrence of ODR. This finding suggests that high preoperative IOP largely affect retinal capillaries via pressure-induced trauma. When IOP is reduced following surgery, arterial inflow increases, which can compromise retinal capillaries.^8^ It is possible that a loss of retinal vessel autoregulation occurs for glaucoma patients. If this is the case, retinal capillaries would fill beyond their limits when IOP is acutely reduced, resulting in retinal haemorrhages.^1,8^ Gupta et al^14^ postulated that sudden hypotony results in a scleral deformity and the subsequent shearing of fragile capillaries. It also may be that an acute decrease in IOP distorts the lamina cribrosa and subsequently compresses the central retinal vein.^10,15^ Unfortunately, none of these mechanisms, all of which involve mechanical and vascular etiologies, have been fully elucidated.

Canaloplasty is a relatively new, nonpenetrating procedure performed to treat POAG. This procedure aims to increase physiological aqueous outflow from the anterior chamber through a trabeculo-Descemet's window into Schlemm canal.^16^ Grieshaber et al^9^ reported a sustained, long-term benefit of canaloplasty in black African patients with POAG. This led them to recommend canaloplasty for patients with enhanced wound healing and scar formation and led us to choose canaloplasty for this young 31-year-old patient. In contrast to traditional trabeculectomy, canaloplasty does not require subconjunctival bleb formation or the use of antimetabolites. The most frequent intraoperative complication was the creation of a false-passage creation (3.3%), and postoperative complication was mild hyphema (45.5%), respectively.^9,17^ Some authors have suggested that average IOP following canaloplasty is not low enough. Therefore, canaloplasty should be considered for patients with mild-to-moderate glaucoma.^18,19^ In the case presented here, there was no evidence during surgery of an obstruction or false-passage creation. In our case, canaloplasty sufficiently lowered IOP in a patient with high IOP (>40 mm Hg) that was not controllable with medication. Immediately following surgery, IOP was very low (7 mm Hg), which partially resulted from a postoperative choroidal detachment. In our case, both preoperative IOP (60 mm Hg at its peak) and the acute IOP change that occurred in 1 day played a role in the development of ODR. Therefore, it is of great importance for physicians to be aware of ODR in patients with severely elevated IOP who planned to undergo canaloplasty.

In conclusion, the current case demonstrates that ODR can occur following canaloplasty. In order to minimize the risk of this rare complication, IOP should be decreased as much as possible before surgery and gradually lowered during surgery in eyes with severely elevated preoperative IOP.

## References

[1] Fechtner RD Minckler D Weinreb RN Complications of glaucoma surgery. Ocular decompression retinopathy. *Arch Ophthalmol* 1992; 110:965–968. 1637282 10.1001/archopht.1992.01080190071032

[2] Mukkamala SK Patel A Dorairaj S Ocular decompression retinopathy: a review. *Surv Ophthalmol* 2013; 58:505–512. 24160727 10.1016/j.survophthal.2012.11.001

[3] Yalvac IS Kocaoglan H Eksioglu U Decompression retinopathy after Ahmed glaucoma valve implantation in a patient with congenital aniridia and pseudophakia. *J Cataract Refract Surg* 2004; 30:1582–1585. 15210243 10.1016/j.jcrs.2003.11.046

[4] Lai JS Lee VY Leung DY Decompression retinopathy following laser peripheral iridoplasty for acute primary angle-closure. *Eye (Lond)* 2005; 19:1345–1347. 15618976 10.1038/sj.eye.6701774

[5] Rao SK Greenberg PB Macintyre RB Ocular decompression retinopathy after anterior chamber paracentesis for uveitic glaucoma. *Retina* 2009; 29:280–281. 18728618 10.1097/IAE.0b013e318185ea54

[6] Kozobolis VP Kalogianni E Katsanos A Ocular decompression retinopathy after deep sclerectomy with mitomycin C in an eye with exfoliation glaucoma. *Eur J Ophthalmol* 2011; 21:324–327. 20872360 10.5301/EJO.2010.5731

[7] Abu Samra K Fernando Sieminski S Sarup V Decompression retinopathy after ExPRESS shunt implantation for steroid-induced ocular hypertension: a case report. *Case Rep Ophthalmol Med* 2011; 2011:303287. 22611508 10.1155/2011/303287 PMC3350207

[8] Alwitry A Khan K Rotchford A Severe decompression retinopathy after medical treatment of acute primary angle closure. *Br J Ophthalmol* 2007; 91:121. 17179132 10.1136/bjo.2006.100479 PMC1857580

[9] Grieshaber MC Pienaar A Olivier J Canaloplasty for primary open-angle glaucoma: long-term outcome. *Br J Ophthalmol* 2010; 94:1478–1482. 20962352 10.1136/bjo.2009.163170

[10] Lee EJ Kim TW Weinreb RN Reversal of lamina cribrosa displacement and thickness after trabeculectomy in glaucoma. *Ophthalmology* 2012; 119:1359–1366. 22464141 10.1016/j.ophtha.2012.01.034

[11] Obana A Gohto Y Ueda N Retinal and subhyaloid hemorrhage as a complication of laser iridectomy for primary angle-closure glaucoma. *Arch Ophthalmol* 2000; 118:1449–1451. 11030835

[12] Mandal AK Jalali S Rao VS Valsalva retinopathy-like hemorrhage associated with combined trabeculotomy-trabeculectomy in a patient with developmental glaucoma. *Ophthalmic Surg Lasers* 2001; 32:330–332. 11475401

[13] Jung KI Lim SA Lopilly Park HY Risk factors for decompression retinopathy after glaucoma surgery. *J Glaucoma* 2014; 23:638–643. 23429610 10.1097/IJG.0b013e318287aba0

[14] Gupta R Browning AC Amoaku WM Multiple retinal haemorrhages (decompression retinopathy) following paracentesis for macular branch artery occlusion. *Eye (Lond)* 2005; 19:592–593. 15319789 10.1038/sj.eye.6701530

[15] Nah G Aung T Yip CC Ocular decompression retinopathy after resolution of acute primary angle closure glaucoma. *Clin Experiment Ophthalmol* 2000; 28:319–320. 11021564 10.1046/j.1442-9071.2000.00325.x

[16] Lewis RA von Wolff K Tetz M Canaloplasty: circumferential viscodilation and tensioning of Schlemm's canal using a flexible microcatheter for the treatment of open-angle glaucoma in adults: interim clinical study analysis. *J Cataract Refract Surg* 2007; 33:1217–1226. 17586378 10.1016/j.jcrs.2007.03.051

[17] Fujita K Kitagawa K Ueta Y Short-term results of canaloplasty surgery for primary open-angle glaucoma in Japanese patients. *Case Rep Ophthalmol* 2011; 2:65–68. 21475647 10.1159/000324808 PMC3072173

[18] Brusini P Canaloplasty in open-angle glaucoma surgery: a four-year follow-up. *ScientificWorldJournal* 2014; 2014:469609. 24574892 10.1155/2014/469609 PMC3915493

[19] Ayyala RS Chaudhry AL Okogbaa CB Comparison of surgical outcomes between canaloplasty and trabeculectomy at 12 months’ follow-up. *Ophthalmology* 2011; 118:2427–2433. 21856008 10.1016/j.ophtha.2011.05.021

